# Teachers should apply the principle of reduction for more sustainable surgical simulation practice: the example of training pharyngolaryngeal surgery in a porcine model

**DOI:** 10.3389/fmed.2023.1226475

**Published:** 2023-08-30

**Authors:** Caroline Payen, Patrice Gallet, Jérôme R. Lechien, Valentin Favier

**Affiliations:** ^1^Faculty of Medicine, Montpellier University, Montpellier, France; ^2^Otolaryngology–Head and Neck Surgery Department, Nancy Regional University Hospital, Lorraine University, Nancy, France; ^3^Nancy-Lorraine School of Surgery, Virtual Hospital of Lorraine (HVL), Lorraine University, Nancy, France; ^4^NGERE Team, INSERM U1256, Lorraine University, Nancy, France; ^5^Otolaryngology–Head and Neck Surgery Department, EpiCURA Hospital, Mons University, Mons, Belgium; ^6^Otolaryngology–Head and Neck Surgery Department, Foch Hospital, Suresnes, France; ^7^Research-Team ICAR, Laboratory of Computer Science, Robotics and Microelectronics of Montpellier (LIRMM), Univ. Montpellier, French National Centre for Scientific Research (CNRS), Montpellier, France; ^8^YO-IFOS Group for Sustainable Development, Young Members of International Federation of Otolaryngology Societies, Paris, France

**Keywords:** surgical simulation, animal model, reduction principle, sustainability, pig, Otolaryngology-head and neck surgery postgraduate degree

## 1. Introduction

Surgical simulation is become increasingly used for surgical training (1), as it provides trainees a controlled learning environment in a safe place for both the patient and the student (2). In most countries, simulation is now a mandatory teaching modality during the residency to acquire surgical skills. At the same time, awareness of climate change (3, 4) is challenging us to find more sustainable educational solutions (4). However, the concept of an environmentally friendly learning is not well established in the literature. When possible, surgical simulation should avoid the use of animals (5) for ethical and sustainable reasons. However, to date, it is not possible to train all technical skills in synthetic or virtual simulators (6), and animal models may still need to be used. As an example, the porcine model allows training on entire organs [e.g.: heart (7), eyes (8)], to train for complex procedures [e.g.: liver transplantation (9), craniostenosis treatment (10), endoscopic submucosal dissection (11), and to design (12)] or to train for the use of medical devices mainly in the field of robotic surgery (10, 13, 14). Given 1/difficulty of accessing food resources around the world (15), and 2/the environmental impact of pig production (16), we need to question the relevance of using such models for surgical learning.

For instance, in the field of Otolaryngology-head and neck surgery (OHNS) only a few simulators are available to train neck and pharyngolaryngeal surgery (17). Some procedures may be learned with low fidelity reusable synthetic simulators, such as percutaneous tracheotomy (18). However, for more complex procedures, few simulation-based teaching solutions are available apart from human cadaver training (17), due to the difficulty of replicating the physical properties of soft tissues (6). A neck surgery simulator must therefore be able to replicate the anatomy and its planes, to be dissectible (19), and to provide haptic sensations close to those encountered in humans (20). The porcine model meets most of these expectations. When used alive (21), it allows the simulation of bleeding, which makes it particularly interesting for learning vascular dissection. In our experience, the use of a dead porcine model provides satisfactory anatomical and haptic fidelity for surgical simulation of the central neck compartment, while diminishing the environmental costs, handling, paperwork, and ethical issues of using live animals. It is thus possible to simulate most surgical procedures on the upper airway, such as tracheotomy (21–23), cricothyroidotomy (24), laryngotracheoplasty (23), total laryngectomy (21) or even some endolaryngeal surgical procedures (25).

In our opinion, when the use of an animal model for surgical simulation is strictly necessary, every effort should be made to optimize the use of the animal so that the greatest number of trainees benefit while limiting the environmental impact of surgical education.

## 2. Ethical considerations

**Figure 1:**
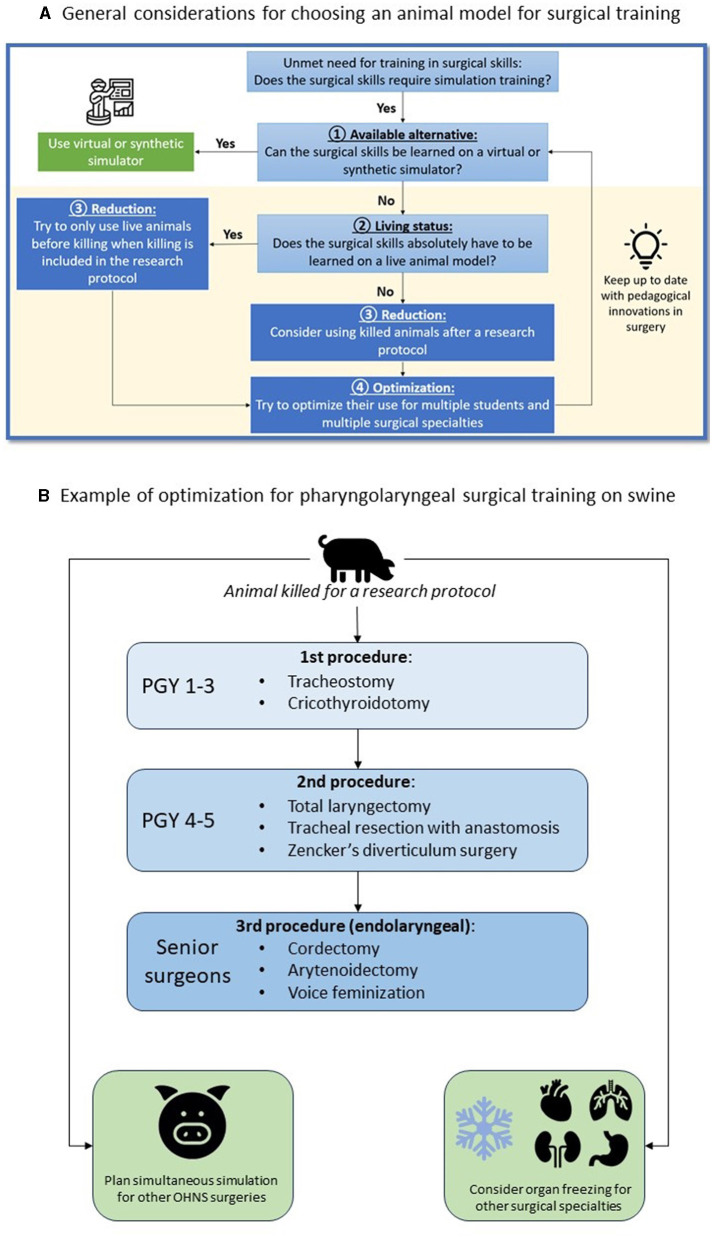
Strategy to reduce and optimize **(A)** the use of animal models in surgical simulation and example with the use of a porcine model for OHNS surgical simulation **(B)**.

Ethical considerations in animal research are based on the “3 R” principles (26):

- The Replacement principle consists in avoiding animal use when it's possible. In the case of pharyngolaryngeal surgery simulation, there is to date no other alternative model (Figure 1).
- The Reduction principle emphasizes the need to reduce animal numbers by optimizing the experimental design.
- The Refinement principle emphasizes the methods to minimize animals suffering and improve wellbeing before and during the experiments.

The use of dead animals from animal research when killing is included in the protocol, responds to the Reduction and Refinement principles. Institutional approval was obtained from the French ministry of higher education, research and innovation (No. APAFIS#26921-202008181721597, approval 2020-066), which has the jurisdiction to provide animal ethics approval.

## 3. Methods

We propose to use the example of pig's median compartment neck surgery to illustrate the procedures that can be trained and tips to optimize its use for education in OHNS and other surgical specialties (Figure 1). First, we advocate to use only dead animals who were killed for a research protocol. If the animal's body is available in the morning, the entire simulation sequence can be conducted. In other situations, the body can be stored outdoors for around 12 h, or frozen for a future use. In the latter case, defrosting takes around 24 h. Ideally, the simulation session has been planned in advance with different surgical specialties in order to make the best use of the available resources. The order of procedures and the rotation of learners of different level must be defined before the simulation session.

### 3.1. Simulation steps

#### 3.1.1. Knowing the anatomical specificities of the central neck compartment in the pig

While the relative dimensions and anatomy of the pharyngolaryngeal-tracheal axis of the pig are similar to humans, some differences should be known and teach before performing procedures (22, 27). This information is provided during the briefing preceding simulation-based learning. It aims at saving time for trainees and allows to complete all the procedures during the simulation session.

#### 3.1.2. Installation

Once the pig has been used alive for research or teaching purposes, and when killing is included in the protocol, it is possible to make use of the animal's body for the simulation in OHNS (Figure 1). The pig should be placed in a supine position to provide cervical extension. Since the larynx is larger in its vertical dimension in pigs than in humans, learners need to palpate and mark on the skin the landmarks of the hyoid bone, thyroid and cricoid cartilage before starting the procedure. A vertical midline incision is preferred to remain in the central compartment. We propose here to describe a surgical simulation session for the sequential learning of tracheotomy, total laryngectomy, and endolaryngeal procedures, to allow three couple of learners of different levels to train on the same animal.

### 3.2. Sequence of simulation and evaluation of learners

For the simulation of neck surgery, we propose a pair made up of one young (postgraduate year 1 or 2, PGY1-2) and one experienced (PGY3-5) resident. First, it is necessary to recall the anatomical differences concerning the procedures to be performed. The PGY1-2 resident will start the simulation by performing a tracheotomy, assisted by PGY3-5. Then, the PGY3-5 resident will perform a total laryngectomy, assisted by PGY1-2. All the steps of a laryngectomy in humans can be performed on the dead pig. To study the oncology principle of resection, a tumor can be simulated using povidone-iodine gel with submucosal injection before entering the pharynx, to assess the surgical margins. The removed larynx will then be used to study endo-laryngeal techniques (cordectomy, arytenoidectomy, or even feminization techniques) during the same simulation session, or later by freezing the larynx. The endolaryngeal surgical simulation allows performing cordectomy, arytenoidectomy, or even feminization techniques. When the simulation is used as a summative evaluation for residents, the teacher will supervise the simulations by giving the different surgical steps without providing spontaneous help. In this way, the procedure can be scored based on the “O-SCORE” scale (27, 28), which allows for the assessment of the resident's ability to perform the procedure independently and safely. At the end of the simulation session, the teacher will debrief the session in the presence of all residents, and the animal body may be used for simulation in other surgical specialties.

## 4. Discussion

According to our experience in three French academic centers, teaching and learning neck surgery on dead pigs provides many advantages. First of all, pig is the only simulator—excepting the human cadaver—allowing performing complete neck procedures, “from skin to skin,” with haptic sensations imitating those of a living tissue. This advantage is always perceived within 12 to 24 h after the killing, bringing a possibility of saving pigs (reduction principle) by coupling with other research manipulations. The main disadvantage is the absence of active bleeding, but massive hemorrhage is uncommon during such surgical procedures in humans. The use of live pigs for tracheostomy (21, 23, 29) or laryngectomy (21) has proved its content [i.e., experts' assessment of the suitability of the pig as a teaching tool (30)] validity. This validity was assessed by sending questionnaires to the experts, asking them to judge whether all the surgical steps could be performed on the model. In our opinion, content validity does not depend on whether the pig is alive or dead to simulate pharyngolaryngeal surgery. Furthermore, some teams already used *ex vivo* porcine skin, larynx, and trachea to simulate tracheostomy (31), cricothyroidotomy (22, 32–34) or partial laryngectomy (35, 36). Using the whole body of a dead pig, as we propose, allows reproducing all the dissection steps.

Secondly, the porcine model provides an anatomy close to that of humans in the medial neck compartment. The anatomy differs in the lateral compartments due to arterial and venous vascularization and lymphatic drainage. Thus, contrary to Alcalá Rueda et al. (22), we do not recommend the use of the pig as a training model for neck lateral dissection procedures. Moreover, the optimized use of the same pig allows performing emergency procedures (tracheostomy, cricothyroidotomy), neck and laryngeal surgery, so that students of different levels can train during the same simulation session.

Finally, other OHNS procedures can be performed in a delayed manner by freezing the pig's head, and other surgical specialties can train from the same animal (Figure 1). This optimization allows for a thoughtful use of the porcine model, as animal dissection is a current ethical issue (3, 8) and may have an environmental impact (16). The use of a previously killed animal for a research protocol requires coordination between research and teaching teams in order to make the best possible use of the animal. We advocate avoiding the use of additional animals for education only, to limit the environmental impact of surgical education.

## 5. Future research

Our feeling is that the use of a dead pig allows to simulate many surgical procedures, without the need to kill an animal only for the purpose of surgical training. This would make it possible to combine quality surgical training with sustainable objectives. To verify this, it will be necessary to prove that the dead pig—as well as the live one—achieves content validity and that it can also prove its ability to help students progress in surgical skills (content validity). Finally, the question of the environmental impact of surgical simulation must be raised. Studies comparing the environmental impact of different learning methods need to be undertaken.

## 6. Conclusion

When designing a curriculum for simulation-based surgical training, teachers should consider both ethical, and environmental aspects. We took the example of the dead porcine model which seems to be a reliable simulator to train midline neck procedures by providing haptic sensation and by its anatomical resemblance to humans. The same animal can be used for several OHNS procedures and by other surgical specialties to responds to the reduction and refinement principles that are essential for ethical and sustainable purposes.

## Author contributions

All authors listed have made a substantial, direct, and intellectual contribution to the work and approved it for publication.
